# Molecular glue binding behavior of phosphoantigens to alpaca butyrophilins

**DOI:** 10.1016/j.jbc.2025.108555

**Published:** 2025-04-26

**Authors:** Chang Liu, Simin Yi, Mengting Zhang, Chun-Chi Chen, Yingle Liu, Zhen Zhang, Rey-Ting Guo, Yunyun Yang

**Affiliations:** 1 State Key Laboratory of Biocatalysis and Enzyme Engineering, Hubei Hongshan Laboratory, School of Life Sciences, Hubei University, Wuhan, PR China; 2 Zhejiang Key Laboratory of Medical Epigenetics, Department of Immunology and Pathogen Biology, School of Basic Medical Sciences, Hangzhou Normal University, Hangzhou, PR China; 3 State Key Laboratory of Virology, College of Life Sciences, Wuhan University, Wuhan, PR China; 4 Zhejiang Provincial Key Laboratory of Applied Enzymology, Yangtze Delta Region Institute of Tsinghua University, Jiaxing, PR China

**Keywords:** phosphantigens, butyrophilins, molecular glue, Vγ9Vδ2 T cell, evolutionary conservation

## Abstract

Vγ9Vδ2 T cells that respond to phosphoantigen (pAg) function as crucial sentinels of the immune system to eradicate pathogen-infected cells and tumor cells. Alpaca (*Vicugna pacos*) is the first nonprimate species identified to possess the pAg-reactive Vγ9Vδ2 T cell subset. However, the molecular mechanism accounting for the pAg recognition of alpaca Vγ9Vδ2 T cells remains unclear. Here, we report the crystal structures of alpaca butyrophilin 3 (*Vp*BTN3) B30.2 domain in complex with the exogenous pAg analog, HMBPP-08, which is a valuable tool for studying the mechanism of butyrophilin-dependent Vγ9Vδ2 T cell activation, and the endogenous pAg analogue, dimethylallyl (S)-thiolodiphosphate (DMASPP). We elucidated that the function of pAgs is governed by their structural differences. Notably, DMASPP acts as a molecular glue in the interaction between the intracellular B30.2 domains of heterologous butyrophilins in alpaca and human. Interestingly, although HMBPP-08 has stronger affinity than DMASPP to *Vp*BTN3 B30.2 domain, HMBPP-08 did not promote heterologous *Vp*BTNs interaction. These findings establish a novel theoretical framework elucidating the mechanisms of Vγ9Vδ2 T cell activation and demonstrate the conserved evolutionary mechanisms underlying cross-species immune adaptation.

γδ T cells play a pivotal role in both adaptive and innate immune responses, with the unique advantage of being able to recognize antigens without requiring major histocompatibility complex restriction, rendering them an promising prospect for cancer immunotherapy (1). The primary subtype of γδ T cells, Vγ9Vδ2 T cells, are activated by the non-peptidic phosphoantigens (pAgs) (2, 3). Isopentenyl pyrophosphate (IPP) was the first pAg to be identified in the early 1990s. IPP and its isomer, dimethylallyl diphosphate (DMAPP), are fundamental components in the mevalonate pathway in both prokaryotes and eukaryotes that are involved in cholesterol synthesis (4, 5). The production of IPP and DMAPP is increased in tumor cells to meet the elevated needs in energy and metabolism, and it has been shown that Vγ9Vδ2 T cells are able to recognize these endogenous mevalonate metabolites (6). In addition to the endogenous pAgs, exogenous pAgs such as (*E*)-4-hydroxy-3-methyl-but-2-enyl pyrophosphate (HMBPP) that are produced by microbes such as *Mycobacterium tuberculosis* and *Listeria monocytogenes* via the nonmevalonate pathway can also activate Vγ9Vδ2 T cells. The activation potency of HMBPP is approximately 1000-fold higher than that of IPP (7, 8).

In 2012, Harly *et al.* identified a butyrophilin (BTN) family member termed butyrophilin 3A1 (BTN3A1) as an essential component in pAg-dependent Vγ9Vδ2 T cell activation in humans (9). BTNs are a subgroup of the immunoglobulin superfamily that shares structural similarity with B7 family costimulatory molecules (9). At present, six BTN-like proteins have been identified, whose members share a multidomain architecture including an extracellular part comprising IgV and IgC domains, a single-pass transmembrane domain, a juxtamembrane coiled-coil domain, an intracellular B30.2 domain and a C-terminal loop in some members (Fig. 1). The binding behavior of BTN3A1 towards pAgs has remained enigmatic for years, until we reported the high-resolution crystal structure of BTN3A1 intracellular B30.2 domain in complex with HMBPP (10). The structural and biochemical experiments were utilized to depict the interaction network of BTN3A1 and pAgs, which offer the experimental supports to the intracellular binding pattern of BTN3A1 toward pAgs (10, 11, 12, 13). In 2020, Herrmann's and Uldrich's groups demonstrated that BTN2A1, the key ligand of Vγ9^+^ T cell receptor (TCR) γ chain, interacts with BTN3A1 in the presence of pAgs to trigger Vγ9Vδ2 T cell activation (14, 15). It has also been shown that the pAg-induced BTN2A1–BTN3A1 interaction depends on the presence of the coiled-coil domain of BTN2A1 but not that of BTN3A1 (16). Furthermore, the extracellular lgV domains from both BTN2A1 and BTN3A1 are not involved in the pAg-mediated interaction (17). More recently, we reported the crystal structure of B30.2 domain of BTN2A1 and BTN3A1 in complex with pAgs, which demonstrates that the BTN3A1–B30.2/pAg complex binds to the BTN2A1-B30.2 homodimer (18). The structure indicates that the pAg binding pocket comprises amino acids from both BTN3A1-B30.2 and BTN2A1-B30.2, making the pAg a molecular glue to form the heterotetramer of BTN3A1 and BTN2A1 (18). The intracellular oligomerization is hypothesized to mediate a spatial distortion that dissociates the extracellular domain of BTN2A1 from that of BTN3A1, which eventually frees the BTN2A1 extracellular domain for the binding of Vγ9^+^ TCR γ chain.

**Figure 1:**
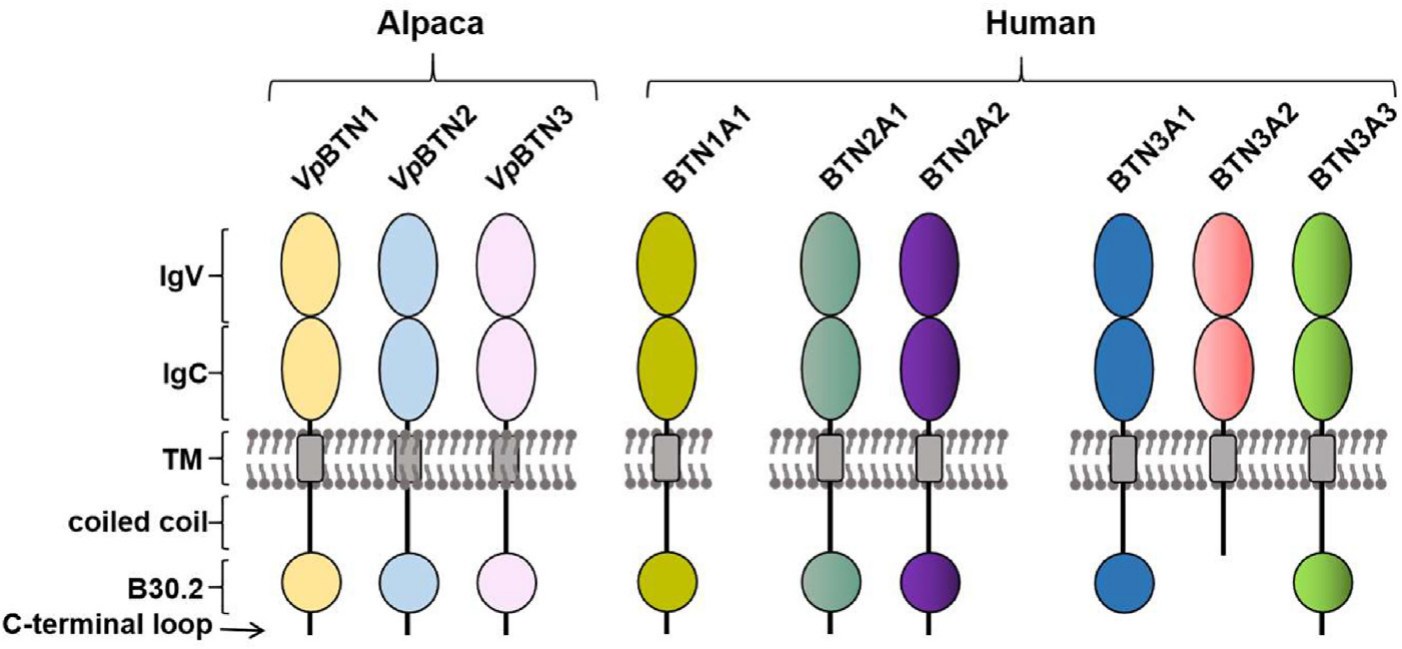
**Schematic representation of the BTN family members in alpaca and human.** BTN, butyrophilin.

Initially, Vγ9Vδ2 T cells were thought to occur exclusively in humans and non-human primates. But the discovery of alpaca (*Vicugna pacos, Vp*) Vγ9Vδ2 T cells overthrows the hypothesis (19). Herrmann's group and our team have recently confirmed that alpaca Vγ9Vδ2 T cells are activated by pAg-binding *Vp*BTN3 (18, 20). *Vp*BTN3 is a member of the alpaca BTN family, which only contains *Vp*BTN1, *Vp*BTN2 in addition to *Vp*BTN3. Given the simplicity of the alpaca family of BTNs compared to the human set of these proteins, alpaca BTNs might represent a useful model to study the activation of Vγ9Vδ2 T cells. Although the domain organization of *Vp*BTNs resembles that of human BTNs (Fig. 1), the interaction between pAg analogs and *Vp*BTNs and *Vp*BTN oligomerization status remain unclear. In this study, we demonstrate that the pAg analogs HMBPP-08 (exogenous) and DMASPP (endogenous) exclusively bind to the B30.2 domain of *Vp*BTN3 but not to that of *Vp*BTN1 or *Vp*BTN2, despite a high sequence identity of approximately 75% shared by these three isoforms. We determined the crystal structures of the *Vp*BTN3 B30.2 in complex with HMBPP-08 and DMASPP, which elucidated the key functional groups driving pAg recognition: OH and methylphenyl groups. Using isothermal titration calorimetry (ITC), site-directed mutagenesis and molecular docking studies, we demonstrate that DMASPP acts as a molecular glue to associate the intracellular B30.2 domains of heterologous BTNs in both alpaca and human. In addition, we discovered that despite HMBPP-08 having a stronger affinity for the *Vp*BTN3 B30.2 domain than DMASPP, it does not facilitate interactions between heterologous *Vp*BTNs. These results should advance our understanding of the molecular mechanisms underlying Vγ9Vδ2 T cell activation.

## Results

### HMBPP-08 binds to the *Vp*BTN3 B30.2 but not to the *Vp*BTN1 or *Vp*BTN2 B30.2 domains

**Figure 2:**
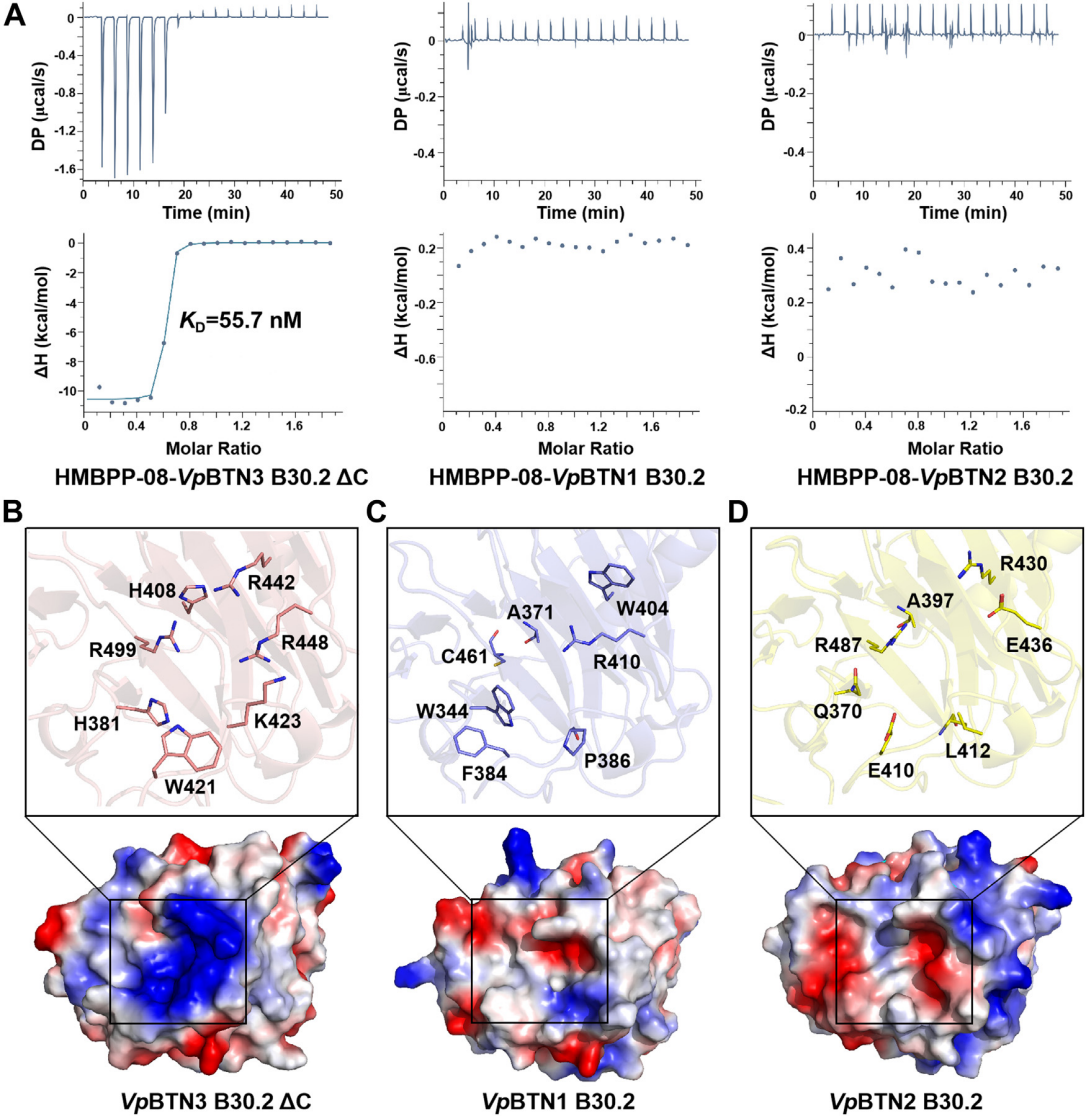
**HMBPP-08 binds to *Vp*BTN3 B30.2 ΔC domain and not to *Vp*BTN1 or *Vp*BTN2 B30.2 domains.***A,* ITC results for HMBPP-08 binding to *Vp*BTN3 B30.2 ΔC domain (*left*), *Vp*BTN1 B30.2 domain (*middle*), and *Vp*BTN2 B30.2 domain (*right*). *B,* electrostatic surface of *Vp*BTN3 B30.2 ΔC structure (*bottom*) and active site residues (*top*). *C,* electrostatic surface of the modeled *Vp*BTN1 B30.2 structure (*bottom*) and the residues corresponding to *Vp*BTN3 B30.2 ΔC (*top*). *D,* electrostatic surface of the modeled *Vp*BTN2 B30.2 structure (*bottom*) and the residues corresponding to *Vp*BTN3 B30.2 ΔC (*top*). The highly cationic region is shown in *blue*. BTN, butyrophilin; HMBPP, (*E*)-4-hydroxy-3-methyl-but-2-enyl pyrophosphate.

HMBPP-08, an HMBPP analog that is valuable in studying the mechanism of BTN-dependent Vγ9Vδ2 T cell activation (Fig. S1), can bind to human BTN3A1 B30.2 domain with higher affinity than HMBPP (10). To investigate how HMBPP-08 binds the *Vp*BTN3 B30.2 domain, we tested the affinity of HMBPP-08 for the *Vp*BTN3 B30.2 domain by ITC assay. Because the presence of the C-terminal loop of *Vp*BTN3 prevents protein crystals from growing, a C-terminal truncated protein (*Vp*BTN3 B30.2 ΔC) was constructed and used in further examinations. Consistent with the results obtained with human BTN3A1 B30.2 domain (10), HMBPP-08 binds to *Vp*BTN3 B30.2 ΔC domain with higher affinity than HMBPP (*K*_D_ = 55.7 nM for HMBPP-08, *K*_D_ = 1.27 μM for HMBPP (18)) (Fig. 2*A*). Considering the high sequence identity among three *Vp*BTNs (Figs. S2 and S3), we also performed ITC assays to determine the binding affinity of HMBPP-08 toward *Vp*BTN1 and *Vp*BTN2 B30.2 domains. Interestingly, HMBPP-08 does not bind to the B30.2 domain of either *Vp*BTN1 or *Vp*BTN2 (Fig. 2*A*).

The efforts to obtain crystals of *Vp*BTN1 and *Vp*BTN2 B30.2 domains failed eventually. Therefore, we simulated the structure of the *Vp*BTN1 and *Vp*BTN2 B30.2 domains with the SWISS-MODEL program, using the structure of *apo*-form *Vp*BTN3 B30.2 ΔC domain (PDB code: 8JYB) as a template. The electrostatic surface of the *apo*-form *Vp*BTN3 B30.2 ΔC structure shows that the pAg-binding site is a positively charged pocket comprising residue W421 and six basic residues (H381, H408, K423, R442, R448, and R499), making it more suitable for binding the negatively charged pyrophosphate group of pAgs (Fig. 2*B*). However, the corresponding residues in *Vp*BTN1 (W344, A371, F384, P386, W404, R410, and C461) form a hydrophobic pocket that is presumed to be unfavorable for the pAg binding (Fig. 2*C*). Similarly, the equivalent region in *Vp*BTN2 that comprises Q370, A397, E410, L412, R430, E436, and R487 also presents an unfavorable environment for pAg binding (Fig. 2*D*). Thus, ITC results and structural analysis suggest that HMBPP-08 only binds to *Vp*BTN3 B30.2 ΔC domain, and not the B30.2 domain of *Vp*BTN1 or *Vp*BTN2.

### Structure of *Vp*BTN3 B30.2 ΔC domain in complex with HMBPP-08

**Figure 3:**
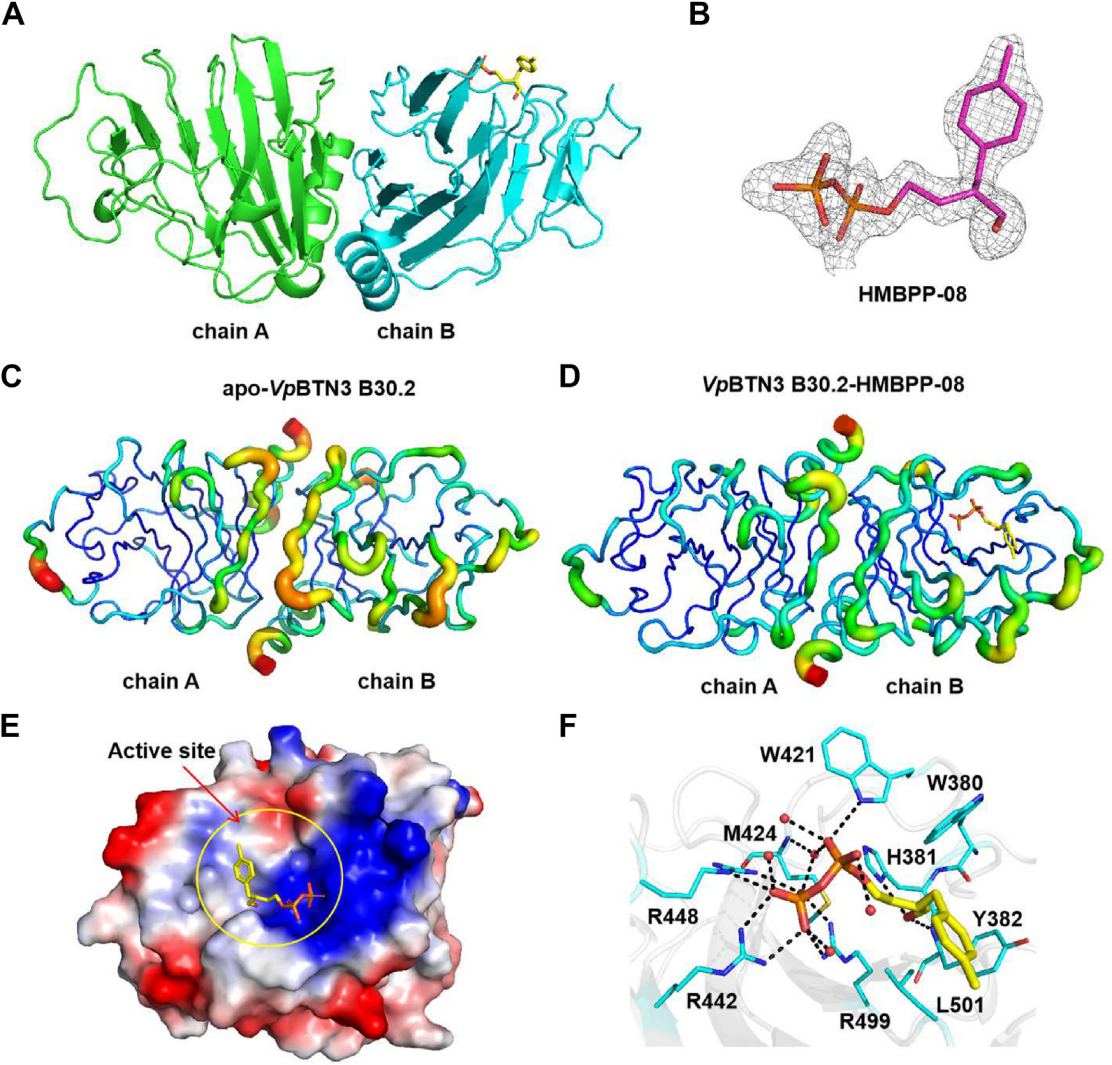
**Structure of the *Vp*BTN3 B30.2 ΔC domain in complex with HMBPP-08.***A,* overall structure of the *Vp*BTN3 B30.2 ΔC domain in complex with HMBPP-08. Two molecules (chain A (*green*) and chain B (*blue*)) in the asymmetric unit are displayed. HMBPP-08 is shown as *stick*. *B,* electron density map of HMBPP-08 in the *Vp*BTN3 B30.2 ΔC/HMBPP-08 complex structure (PDB code: 9LNZ). The *F*_*o*_*-F*_*c*_ omit map of HMBPP-08 in the B chain contoured at 2.5 σ is shown in *gray mesh*. *C,* B-factor putty of (*C*) *ap*o-*Vp*BTN3 B30.2 ΔC and (*D*) *Vp*BTN3 B30.2 ΔC/HMBPP-08 are displayed. *E,* electrostatic potential surface of the B chain of *Vp*BTN3 B30.2 ΔC/HMBPP-08 (*red*, negative charge; *blue*, positive charge). The active site is indicated by a *yellow circle*. *F,* a detailed view of the HMBPP-08 interaction network in *Vp*BTN3 B30.2 ΔC domain with HMBPP-08. HMBPP-08 and protein residues are shown as *sticks* and waters as *red spheres*. *Dashed lines*, distance <3.5 Å. BTN, butyrophilin; HMBPP, (*E*)-4-hydroxy-3-methyl-but-2-enyl pyrophosphate.

To investigate the detailed interactions between HMBPP-08 and *Vp*BTN3 B30.2 ΔC, we obtained the crystal structure of the *Vp*BTN3 B30.2 ΔC domain in complex with HMBPP-08 by soaking HMBPP-08 with the *apo*-form *Vp*BTN3 B30.2 ΔC crystals. *Vp*BTN3 B30.2 ΔC/HMBPP-08 that was crystallized in space group *P*2_1_2_1_2_1_ was refined to a high resolution of 1.92 Å (PDB code: 9LNZ, Table S1). This structure contains two molecules, termed chain A and chain B, in an asymmetric unit (Fig. 3*A*). Chains A and B are highly identical to each other and have a Cα rmsd of 0.176 Å for 166 aligned residues (Fig. S4). Clear electron density maps of HMBPP-08 can only be observed in chain B (Fig. 3*B*). Notably, the B-factor values at residues 401 to 406, 435 to 441, 446 to 456, and 502 to 506 of chain B in the *apo*-form structure are higher than the corresponding positions of chain A (Fig. 3*C*), which were reduced upon the binding of HMBPP-08 (Fig. 3*D*). This implies that HMBPP-08 binding might render local stabilization.

HMBPP-08 binds to a positively charged pocket formed by basic residues (H381, R421, R442, and R448), aromatic residues (W380, Y382, and W421), and a hydrophobic residue (L501) (Fig. 3*E*). The pyrophosphate group of HMBPP-08 forms hydrogen bonds with the side chain of residues R442, R448, R499, W421 and the main chain N of M424 *via* a bridging water molecule (Fig. 3*F*). Meanwhile, the OH group of HMBPP-08 forms hydrogen bonds with the imidazole group of H381 and the main chain N of Y382. Furthermore, the methylphenyl group of HMBPP-08 forms hydrophobic interactions with residues Y382 and L501 (Fig. 3*F*). The binding patterns of HMBPP-08 to *Vp*BTN3 B30.2 ΔC are identical to those observed in our previously reported crystal structure of the BTN3A1 B30.2/HMBPP-08 complex (PDB code: 6J06) (10). Meanwhile, this interaction pattern is consistent with the previously reported finding that variants R442A and H381A completely disrupt *Vp*BTN3 binding to HMBPP (20). Although there is no direct interaction between residue K423 and HMBPP-08, residue K423 forms part of the positively charged pocket, which is conducive to the binding of pAgs (Fig. S5). This structural information explains why variant K423A still retains partial affinity for *Vp*BTN3 B30.2 (20).

### Structural basis for different binding behavior of endogenous and exogenous pAg analogs to *Vp*BTN3

**Figure 4:**
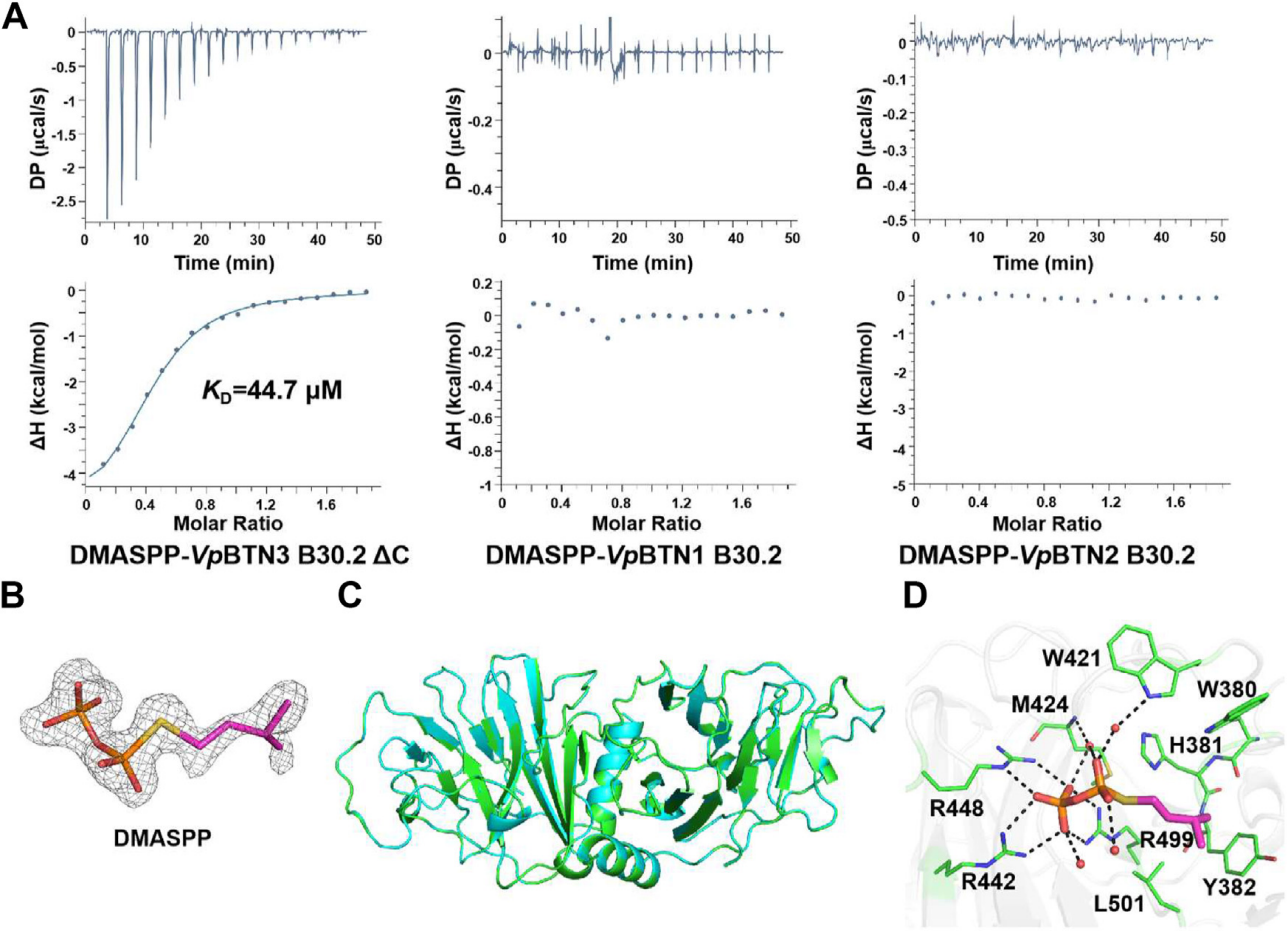
**Binding mode of DMASPP in *Vp*BTN3 B30.2 domain.***A,* ITC results for DMASPP binding to the *Vp*BTN3 B30.2 ΔC domain (*left*), *Vp*BTN1 B30.2 domain (*middle*), and *Vp*BTN2 B30.2 domain (*right*). *B,* electron density map of DMASPP in the *Vp*BTN3 B30.2 ΔC–DMASPP complex structure (PDB code: 9LN2). The *F*_*o*_*-F*_*c*_ omit map of DMASPP in the B chain contoured at 2.5 σ is shown in *gray mesh*. *C,* structure superimposition of *Vp*BTN3 B30.2 ΔC/DMASPP and *Vp*BTN3 B30.2 ΔC/HMBPP-08 (*cyan*; PDB code: 9LNZ). *D,* the detailed view of DMASPP interaction network in *Vp*BTN3 B30.2 ΔC/DMASPP complex structure. Ligand, protein residues, and water molecules are presented as in Fig. 3*F*. *Dashed lines*, distance <3.5 Å. BTN, butyrophilin; HMBPP, (*E*)-4-hydroxy-3-methyl-but-2-enyl pyrophosphate; DMASPP, dimethylallyl (S)-thiolodiphosphate; ITC, isothermal titration calorimetry.

The ITC results showed weaker affinity between DMASPP, a DMAPP analog (Fig. S1), and *Vp*BTN3 B30.2 ΔC domain than HMBPP-08. Moreover, we also confirmed that DMASPP does not interact with *Vp*BTN1 or *Vp*BTN2 B30.2 domains (Fig. 4*A*). To investigate the different binding affinities between two pAg analogs and the *Vp*BTN3 B30.2 ΔC domain, we obtained the complex structure of *Vp*BTN3 B30.2 ΔC/DMASPP at 1.77 Å resolution (PDB code: 9LN2, Table S1). Similar to *Vp*BTN3 B30.2 ΔC/HMBPP-08 complex, the *Vp*BTN3 B30.2 ΔC/DMASPP structure contains two molecules in an asymmetric unit, and the electron density maps of DMASPP are exclusively observed in chain B (Fig. 4*B*). Notably, the electron density map of the isoprenyl moiety of DMASPP observed here is clearer than that of DMAPP in the *Vp*BTN3 B30.2 ΔC/DMAPP complex reported recently (18). Thus, the DMASPP bound in the *Vp*BTN3 B30.2 ΔC complex represents concrete evidence of the pAg binding pose. Alignment with the *Vp*BTN3 B30.2 ΔC/HMBPP-08 structure yields a rmsd of 0.069 Å for 354 Cα pairs (Fig. 4*C*), which suggests a similar overall structure of two complex structures. However, detailed interaction analysis revealed that no hydrogen bonding interaction from H381 and Y382 was observed owing to the lack of an OH group in DMASPP (Fig. 4*D*). Moreover, due to the absence of methylphenyl groups on DMASPP, the hydrophobic interactions with hydrophobic residues around the active pocket are absent in the *Vp*BTN3 B30.2 ΔC/DMASPP structure (Fig. 4*D*). This deficiency of functional groups in DMASPP results in weaker binding, which is consistent with the precise interactions between the *Vp*BTN3 B30.2 ΔC domain and the pAg analogs observed in the crystal structures.

### The DMASPP-mediated interaction between the intracellular B30.2 domains of *Vp*BTN2 and *Vp*BTN3

**Figure 5:**
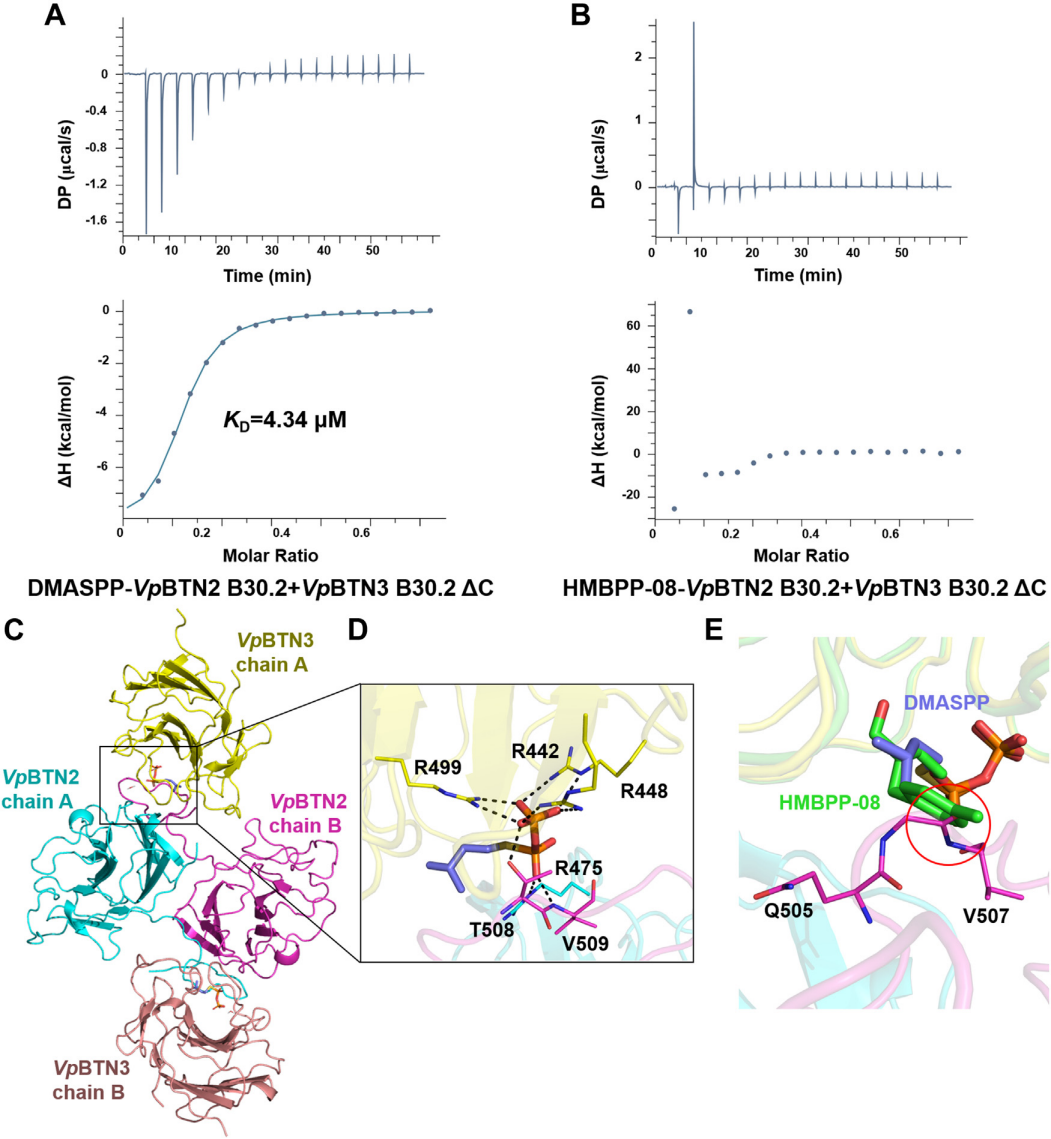
**DMASPP acts as molecular glue in the interaction between the intracellular B30.2 domains of *Vp*BTN2 and *Vp*BTN3.***A,* ITC result shown that DMASPP promotes interaction between *Vp*BTN2 B30.2 and *Vp*BTN3 B30.2 ΔC in first titration mode. *B,* ITC result for HMBPP-08 binding to *Vp*BTN2 B30.2 domain and *Vp*BTN3 B30.2 ΔC domain in first titration mode. *C,* model of *Vp*BTN3-DMASPP-*Vp*BTN2 B30.2 structure. *D,* the zoom-in view of DMASPP-binding site. Ligand and protein residues are presented as in Fig. 3*F*. *Dashed lines*, distance <3.5 Å. *E,* the structural alignment of *Vp*BNT3/HMBPP-08 and *Vp*BTN2–DMASPP–*Vp*BTN3 B30.2 complex model. The *red circle* serves to highlight the steric hindrance between HMBPP-08 and the loop region of *Vp*BTN2 B30.2. BTN, butyrophilin; HMBPP, (*E*)-4-hydroxy-3-methyl-but-2-enyl pyrophosphate; DMASPP, dimethylallyl (S)-thiolodiphosphate; ITC, isothermal titration calorimetry.

Next, we proceeded to investigate whether HMBPP-08 and DMASPP could also promote interactions between the intracellular B30.2 domains of heterologous *Vp*BTNs. This event was probed by two titration trials. One utilized pAg analogues to titrate a mixture of *Vp*BTN1 or *Vp*BTN2 and *Vp*BTN3 B30.2 ΔC domains and the other employed *Vp*BTN1 or *Vp*BTN2 B30.2 domain to titrate *Vp*BTN3 B30.2 ΔC domain and pAg analogs preformed complex. ITC experiments confirmed that DMASPP facilitates the association between the B30.2 domain of *Vp*BTN2 and *Vp*BTN3, with a *K*_D_ of 4.34 μM for the first titration trial and *K*_D_ of 22.8 μM for the second titration trial (Figs. 5*A* and S6*A*). The results revealed that as an analog of DMAPP, DMASPP enhanced the interaction between *Vp*BTN3 and *Vp*BTN2 B30.2 domains twice as effectively as DMAPP (*K*_D_ = 46.5 μM for the second titration trial (18)). In addition, as the full-length *Vp*BTN3 B30.2 domain with *K*_D_ of 15.2 μM for the second titration trial, C-terminal truncated *Vp*BTN3 B30.2 domain did not influence the affinity (Fig. S6*B*). However, this was not observed for the interaction between the *Vp*BTN1 B30.2 domain and the *Vp*BTN3 B30.2 ΔC domain in the presence of DMASPP (Fig. S6*C*). Unexpectedly, HMBPP-08 did not promote the binding between B30.2 domains of *Vp*BTN2 and *Vp*BTN3, even with its robust affinity for the *Vp*BTN3 B30.2 ΔC domain (Figs. 5*B* and S6*D*).

We intended to solve the ternary structure of *Vp*BTN2 and *Vp*BTN3 B30.2 domain in complex with DMASPP to elucidate the different behavior of two pAg analogs in mediating the *Vp*BTNs interactions. Unfortunately, our efforts in obtaining the complex crystals eventually failed. In our previous study, we successfully obtained the structure of *Vp*BTN2 and *Vp*BTN3 B30.2 domains complexed with a high-affinity HMBPP (18). Therefore, we performed molecular docking experiments to obtain the binding mode of DMASPP, utilizing the *Vp*BTN2-HMBPP-*Vp*BTN3 B30.2 structure (PDB code: 8HJT) as a reference (Fig. 5*C*). In the *Vp*BTN2-DMASPP-*Vp*BTN3 B30.2 structure model, DMASPP underwent interaction to *Vp*BTN3 B30.2 ΔC domain in a same manner to that was observed in the *Vp*BTN3 B30.2 ΔC-DMASPP structure (Fig. 5*D*). Moreover, the pyrophosphate group of DMASPP forms hydrogen bond interactions with residue R475 from chain A and residues T508 and V509 from chain B of *Vp*BTN2 B30.2 (Fig. 5*D*). To verify the structural model, we conducted mutagenesis to generate three variants of *Vp*BTN2 B30.2: R475A, T508M, and V509A (Fig. S7 and Table S2). According to the ITC results, variants R475A and T508M showed no affinity to the *Vp*BTN3 B30.2 ΔC and DMASPP preformed complex, and variant V509A exhibited a reduced affinity (*K*_D_ = 44.2 μM, Fig. S8). Taking together the ITC results, structural modeling and mutagenesis experiments, DMASPP may act as a molecular glue to facilitate the interaction between the B30.2 domains of *Vp*BTN2 and *Vp*BTN3 *via* the aforementioned interaction networks.

We next tried to dock HMBPP-08 in a similar manner to obtain a structural model of *Vp*BTN2-HMBPP-08-*Vp*BTN3 B30.2 but found that molecular docking cannot be executed. Aligning *Vp*BNT3/HMBPP-08 and *Vp*BTN2-DMASPP-*Vp*BTN3 B30.2 structure indicate that the bulky methylphenyl moiety of HMBPP-08 would clash with the loop region consisting of residues 505 to 507 of *Vp*BTN2 B30.2 domain, which prevents HMBPP-08 from associating the *Vp*BTN2 B30.2 domain to the *Vp*BTN3 B30.2 domain (Fig. 5*E*). These observations are similar to the previously reported results that HMBPP-08 is incapable of inducing the association of human BTN3A1 and BTN2A1 B30.2 domain (18).

### Parallels between DMASPP molecular glue function in human and alpaca

To investigate whether the molecular glue function of DMASPP in alpaca BTNs translatable to human BTNs, we carried out an ITC assay to evaluate the affinity between human BTN3A1 and BTN2A1 B30.2 domains in the presence of DMASPP. The results of the two titration trials demonstrated that DMASPP promotes the interactions of BTN3A1 and BTN2A1 B30.2 domains, with *K*_D_ values of 8.19 μM and 18.8 μM, respectively (Fig. S9). These results suggest that the endogenous pAg analog, DMASPP, is responsible for the interaction between heterologous BTNs in primates and nonprimates, confirming the conservation of pAg-binding BTNs in activating Vγ9Vδ2 T cells.

## Discussion

Our early research has taken significant strides toward elucidating the structural dynamics and biochemical mechanisms underlying the activation of human Vγ9Vδ2 T cells by pAgs, contributing substantially to the field of cancer immunotherapy. We have provided vital insights into the role of endogenous and exogenous pAgs in the complex molecular interplay involving BTN3A1, BTN2A1, and the TCR of Vγ9Vδ2 T cells, an interaction pivotal to Vγ9Vδ2 T cell activation (18). It is interesting to note that the Vγ9Vδ2 T cell activation, previously believed to be exclusive to humans and higher primates, was also observed in alpacas. This groundbreaking discovery by the Herrmann's group (20) has encouraged us to further explore the activation mechanism of Vγ9Vδ2 T cells across different species. In this context, our findings that endogenous and exogenous pAgs function as a molecular glue to promote heteromeric association between the intracellular domains of *Vp*BTN3 and *Vp*BTN2 provide novel insights and expand our understanding of the complexity and versatility of immune response mechanisms (18).

This study elucidates the mystery behind the selective recognition of pAg analogs by *Vp*BTNs. For instance, we discovered that despite *Vp*BTNs have high sequence identity, *Vp*BTN1, *Vp*BTN2, and *Vp*BTN3 demonstrate distinct binding affinities toward the pAg analogs HMBPP-08 and DMASPP. Our ITC and molecular docking studies have demonstrated that the DMASPP functions as a molecular glue, enabling the interaction between the intracellular B30.2 domains of *Vp*BTN2 and *Vp*BTN3. In addition, we discovered that despite the stronger affinity of HMBPP-08 for the *Vp*BTN3 B30.2 ΔC domain, HMBPP-08 fails to facilitate interactions between *Vp*BTNs. From our structural model of *Vp*BTN2-HMBPP-08-*Vp*BTN3 B30.2, the bulky substituents of HMBPP-08 could not accommodate the loop region comprising residues 505 to 507, consequently preventing it from promoting the interactions between *Vp*BTN2 and *Vp*BTN3 B30.2 domains. It is consistent with the observation that HMBPP-08 is unable to facilitate the association between human BTN3A1 and BTN2A1 B30.2 domains (18). The aforementioned property of HMBPP-08 suggests its potential to be regarded as an inhibitor of human γδ T cell function. The ITC results indicated that DMASPP could also function as a molecular glue between human BTN3A1 and BTN2A1 B30.2 domains. Glycosylation modifications of *Vp*BTNs were dispensable for this functional interaction, aligning with previous findings in human BTN protein studies (15). These findings highlight a conservation of pAg-mediated activation of Vγ9Vδ2 T cells across primates and nonprimates. DMASPP's properties suggest its potential to act as an activator of human γδ T cell function. However, it is important to remember that our findings are based on *in vitro* studies and need to be validated *in vivo* to truly appreciate the functional implications in a physiological context.

In conclusion, this study has significantly enhanced our understanding of the intricate mechanisms regulating Vγ9Vδ2 T cell activation. Our findings shed light on the role of pAgs as the molecular glue in promoting the heteromeric association of BTN family proteins, thereby elucidating a crucial aspect of immune response, particularly in the context of immunotherapy. These insights could potentially pave the way for the development of more effective immunotherapeutic strategies against cancer and infectious diseases.

## Experimental procedures

### Chemicals

HMBPP-08 was synthesized as described previously (10). DMASPP were purchased from Echelon.

### Protein expression and purification

The genes encoding *Vp*BTN1 B30.2 (residues 285–526), *Vp*BTN2 B30.2 (residues 311–535), and *Vp*BTN3 B30.2 ΔC (residues 323–513) were chemically synthesized and subcloned into pET32a vector with *Nco*I and *Xho*I restriction sites by Wuhan GeneCreate Biological Engineering Co., Ltd. The recombinant plasmids were individually transformed into *Escherichia coli* BL21(DE3) strains for protein expression. All BL21(DE3) strains harboring the specified recombinant plasmids were cultured in Luria-Bertani medium at 37 °C until the A_600_ reached 0.6 and then induced by adding 0.5 mM IPTG at 25 °C for 18 h. The induced strains were harvested by centrifugation at 6000 *g* for 15 min, and subsequently resuspended in a lysis buffer containing 25 mM Hepes pH 7.5, 150 mM NaCl and 20 mM imidazole. Strains were lysed using a French press and debris was removed by centrifugation at 20,000 *g* and 4 °C for 1 h. The supernatant was applied to an AKTA purifier fast protein liquid chromatography system coupled with a Ni-nitrilotriacetic acid (NTA) column (GE Healthcare). The recombinant target proteins were dialyzed against a buffer containing 25 mM Hepes pH 7.5 and 150 mM NaCl, and subjected to the Tobacco Etch Virus protease digestion overnight to remove the 6 × His tag on the N terminal. The mixture was then passed through a Ni-NTA column to remove the His-tagged portion, with the untagged protein eluted in the imidazole-free buffer. All *Vp*BTN2 B30.2 domain mutants were generated using a standard PCR mutagenesis strategy, overexpressed, and purified following the same procedures as for the WT protein. The purity of the proteins was verified by SDS-PAGE analysis and the purified proteins were stored at −80 °C for crystallization screening and further analyses.

The recombinant plasmid of BTN2A1 B30.2 was subcloned into pET28a vector with *NcoI* and *XhoI* restriction sites. Protein expression was induced by 0.2 mM IPTG at 16 °C for 24 h. Subsequently, BTN2A1 was purified with a Ni-NTA column (GE Healthcare) and DEAE Sepharose column. BTN3A1 B30.2 was prepared following the same expression and purification procedures as those used for *Vp*BTN3 B30.2.

### Crystallization and structure determination

All crystals were obtained using the sitting-drop vapor diffusion method. In general, 1 μL of target proteins were mixed with 1 μL of reservoir solution in 48-well Cryschem plates and equilibrated against 100 μL reservoir solution. The optimal crystallization condition of *apo*-*Vp*BTN3 B30.2 ΔC was 0.1 M Bis-Tris pH 5.5, 2.0 M ammonium sulfate. The complex crystal structures of *Vp*BTN3 B30.2 ΔC-HMBPP-08 and *Vp*BTN3 B30.2 ΔC-DMASPP were solved by soaking the *apo*-*Vp*BTN3 B30.2 ΔC crystals with HMBPP-08/DMASPP. Prior to data collection, the soaked crystals were cryoprotected in the mother liquor supplemented with 15% PEG 200, followed by flash freezing. The X-ray diffraction data were obtained at the in-house beamline Bruker D8 Venture coupled with a CMOS-PHOTON III detector at Hubei University. The X-ray diffraction datasets were initially processed with PROTEUM3, the structures were solved by molecular replacement method with the BTN3A1 B30.2 structure (PDB code: 4N7I) as the search model. Further model adjustment and refinement were conducted by using Refmac5 (https://www.ccp4.ac.uk/html/refmac5.html), PHENIX (https://phenix-online.org), and Coot (https://www2.mrc-lmb.cam.ac.uk/personal/pemsley/coot/) (21, 22, 23). All graphics for structures were prepared using the PyMOL program (http://pymol.sourceforge.net/).

### ITC assays

ITC experiments were carried out at 25 °C using a MicroCal PEAQ-ITC instrument (GE Healthcare). The protocol involved an initial injection of 0.4 μL, followed by 19 subsequent injections (each 2 μL) at intervals of 150 s. The buffer used for sample preparation was composed of 25 mM Hepes pH 7.5, 150 mM NaCl.

For the assessment of HMBPP-08 binding to the *Vp*BTN1 B30.2 domain, *Vp*BTN2 B30.2 domain, *Vp*BTN3 B30.2 ΔC domain, the sample cell and injection syringe were filled with the buffer containing 100 μM of protein and 1 mM of HMBPP-08, respectively. To detect the combination between DMASPP and the aforementioned proteins, ITC experiments were performed with a 10:1 ratio of DMASPP (4 mM) to the B30.2 domain (400 μM).

For experiments assessing the binding of pAgs to the *Vp*BTN3 B30.2 ΔC domain with *Vp*BTN2 B30.2 domain, *Vp*BTN2 B30.2 domain and *Vp*BTN3 B30.2 ΔC domain were incubated in the buffer at a 2:1 ratio and loaded into the sample cell. And the injection syringe was filled with the buffer containing pAgs, with the 1:10 ratio of *Vp*BTN3 B30.2 ΔC to pAgs. ITC experiments of pAgs binding to the BTN2A1 B30.2 and BTN3A1 B30.2 complex followed the same procedures as those used for the *Vp*BTN3 B30.2 ΔC and *Vp*BTN2 B30.2 domain. The used pAgs were DMASPP and HMBPP-08, which were concentrated at 2 mM and 1 mM, respectively, in the above experiment.

For the experiments investigating the binding of *Vp*BTN2 B30.2 domain to the *Vp*BTN3 B30.2 ΔC domain in the presence of pAgs, *Vp*BTN3 B30.2 ΔC domain and pAgs were incubated in the buffer at a 1:10 ratio and loaded into the sample cell. And the injection syringe was filled with *Vp*BTN2 B30.2 domain (including their mutants) at a concentration of 2 mM. The used pAgs in this experiment, DMASPP and HMBPP-08, were concentrated to 2 mM and 1 mM, respectively.

### Docking studies

The docking poses were generated by Glide (22) in Schrodinger suite (Maestro 11.1), using the *Vp*BTN2-HMBPP-*Vp*BTN3 B30.2 structure (PDB code: 8HJT) as the reference. Both DMASPP and HMBPP-08 were prepared, and their ionization states were determined. Hydrogen atoms were added to the proteins of *Vp*BTN2 and *Vp*BTN3 B30.2 and water molecules forming fewer than three hydrogen bonds with nonwater molecules were removed. The prepared proteins were subsequently utilized to generate grids employing default settings. Ultimately, the prepared compounds and proteins were docked using standard precision method in Glide. The highest-scoring docking positions were visualized and analyzed using the PyMOL program.

## Data availability

The atomic coordinates and structure factors of the reported structures have been deposited in the Protein Data Bank under accession codes as follow: 9LNZ and 9LN2. Any additional information required to reanalyze the data reported in this work paper is available from the lead contact upon request.

## Supporting information

This article contains supporting information.

## Conflict of interest

The authors declare that they have no conflicts of interest with the contents of this article.
