# Supplementary material for: Molecular glue binding behavior of phosphoantigens to alpaca butyrophilins
Source: J Biol Chem. 2025 Apr 26;301(6):108555. doi: 10.1016/j.jbc.2025.108555 (PMC12152872; doi:10.1016/j.jbc.2025.108555)
Supplement: Supporting Information [file mmc1.docx]

Molecular glue binding behavior of phosphoantigens to alpaca butyrophilins

Chang Liu^1^, Simin Yi^1^, Mengting Zhang^1^ Chun-Chi Chen^1,4^, Yingle Liu^2^, Zhen Zhang^3^, Rey-Ting Guo^1,4^*, and Yunyun Yang^1^*

^1^State Key Laboratory of Biocatalysis and Enzyme Engineering, Hubei Hongshan Laboratory, School of Life Sciences, Hubei University, Wuhan 430062, PR China.

^2^State Key Laboratory of Virology, College of Life Sciences, Wuhan University, Wuhan 430072, PR China.

^3^Zhejiang Provincial Key Laboratory of Applied Enzymology, Yangtze Delta Region Institute of Tsinghua University, Jiaxing 314006, PR China.

^4^Zhejiang Key Laboratory of Medical Epigenetics, Department of Immunology and Pathogen Biology, School of Basic Medical Sciences, Hangzhou Normal University, Hangzhou 311121, PR China.

*Corresponding authors. E-mail addresses: [guoreyting@hubu.edu.cn](mailto:guoreyting@hubu.edu.cn) (R.-T. G), [yang_yy@hubu.edu.cn](mailto:zhangyonghui@tsinghua.edu.cn) (Y. Y)

**
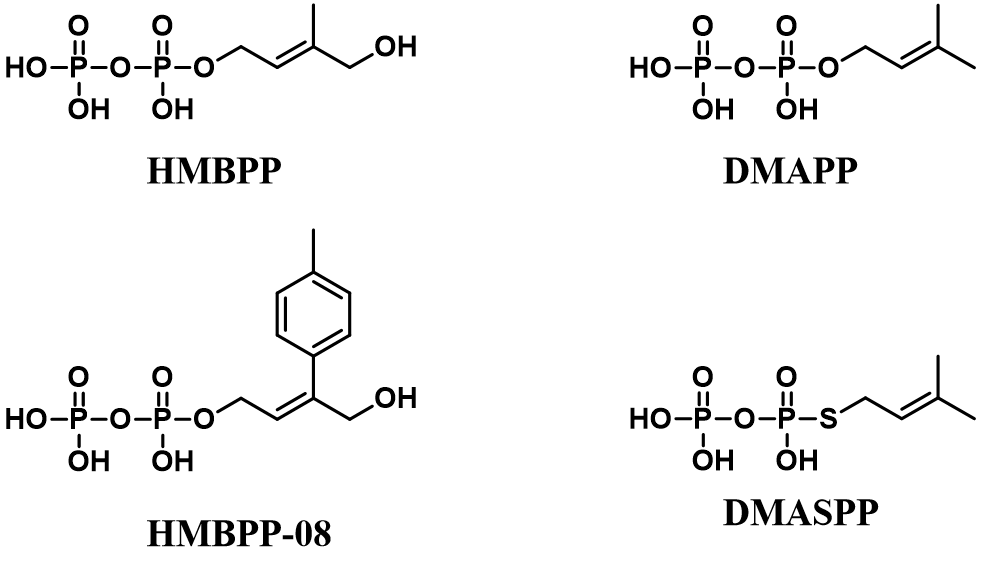
**

**Fig. S1 Chemical structures of pAgs and pAg analogs: HMBPP, HMBPP-08, DMAPP, and DMASPP.**


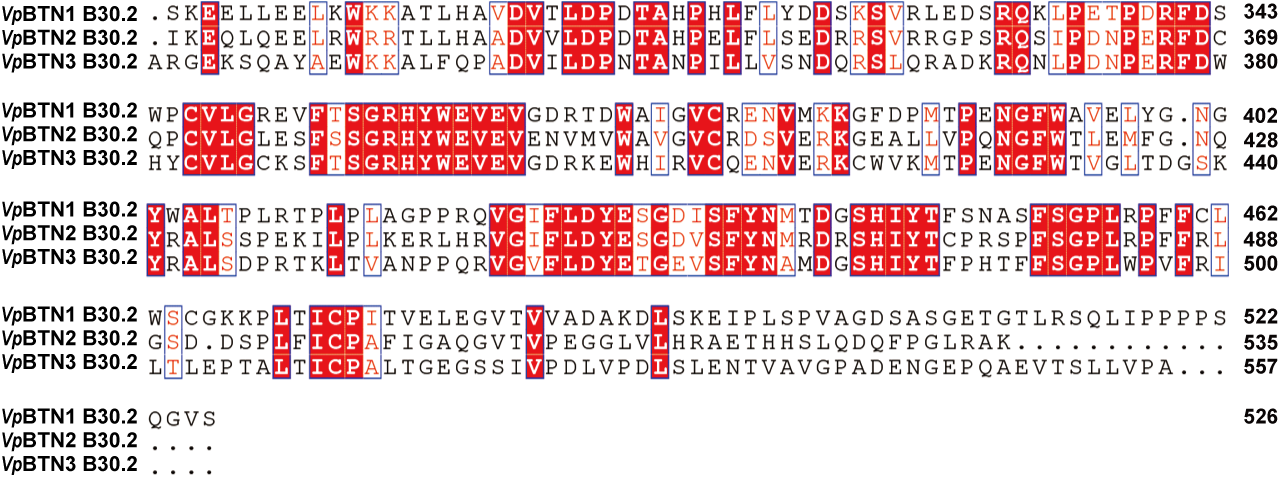


**Fig. S2 Sequence alignment of the B30.2 domains of *Vp*BTN1, *Vp*BTN2 and *Vp*BTN3.**


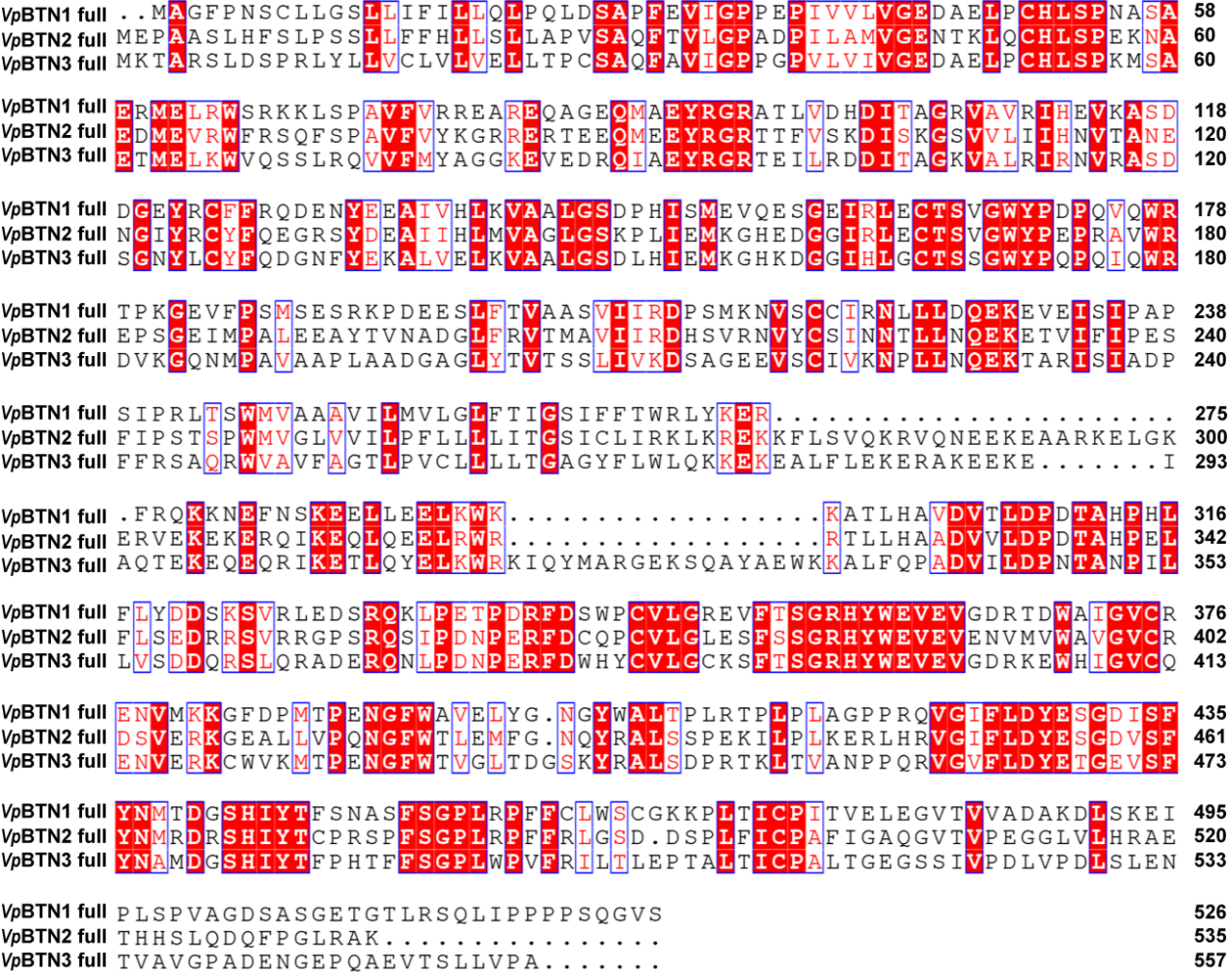


**Fig. S3 Sequence alignment of full-length *Vp*BTN1, *Vp*BTN2 and *Vp*BTN3.**

**
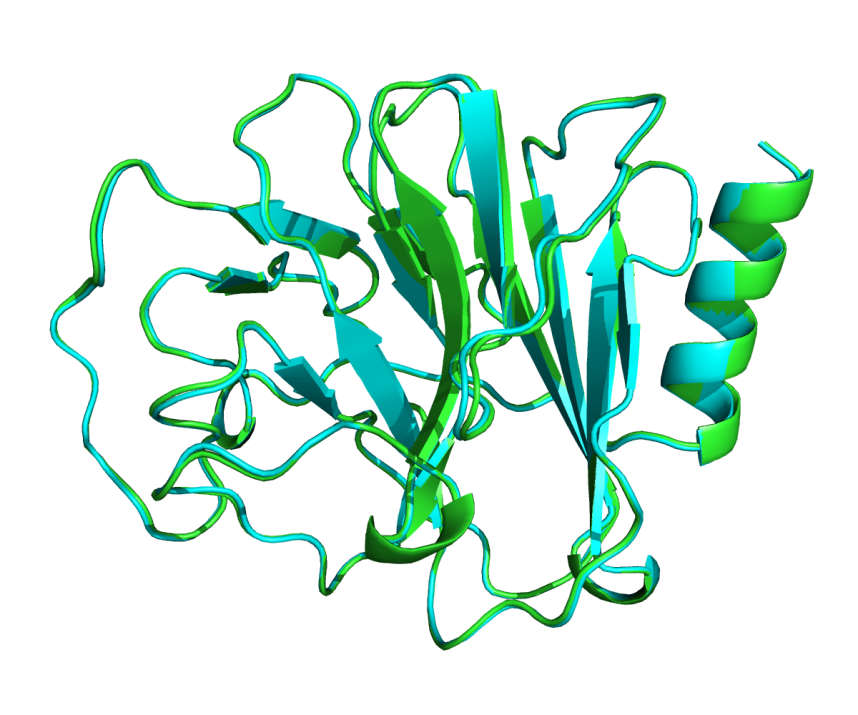
**

**Fig. S4 Structure superimposition of A chain** **(green) and B chain (blue) of *Vp*BTN3 B30.**2 **ΔC/HMBPP-08.**

**
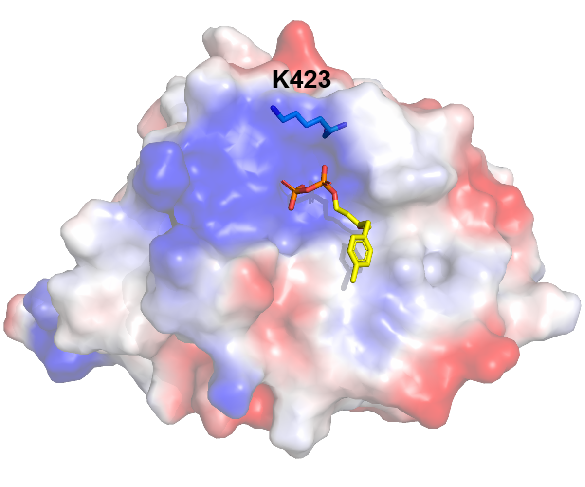
**

**Fig. S5 Electrostatic potential surface of the B chain of *Vp*BTN3 B30.2 ΔC/HMBPP-08**. K423 and HMBPP-08 are shown as sticks.

**
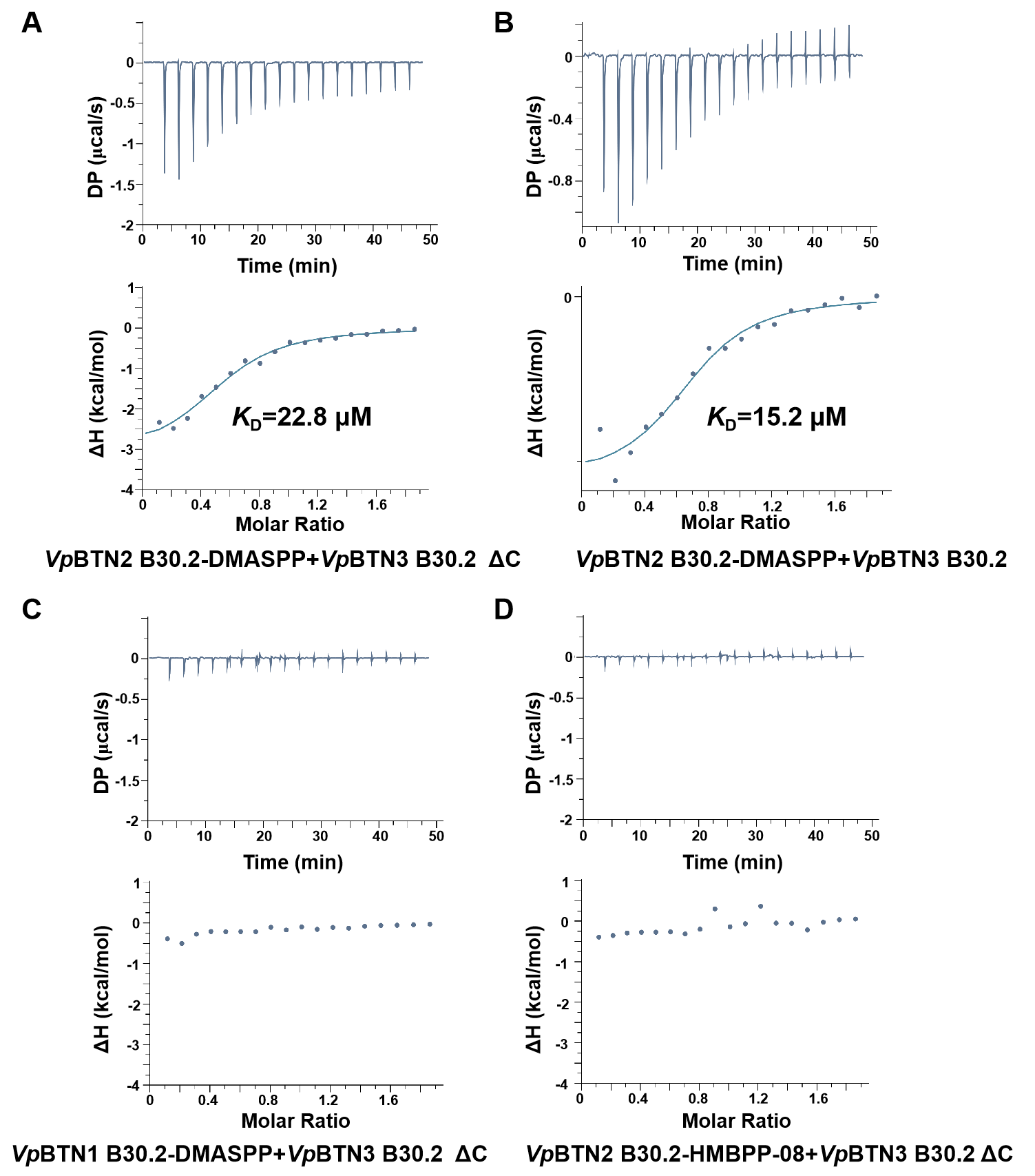
**

**Fig. S6 ITC results for*Vp*BTN1 or *Vp*BTN2 B30.2 domain to titrate the *Vp*BTN3 B30.2 ΔC domain and pAg analogues.**

1. ITC result for *Vp*BTN2 B30.2 domain binding to the *Vp*BTN3 B30.2 ΔC/DMASPP. (B) ITC result for *Vp*BTN2 B30.2 domain binding to the *Vp*BTN3 B30.2/DMASPP. (C) ITC result for *Vp*BTN1 B30.2 domain binding to the *Vp*BTN3 B30.2 ΔC/DMASPP. (D) ITC result for *Vp*BTN2 B30.2 domain binding to the *Vp*BTN3 B30.2 ΔC/HMBPP-08.

**
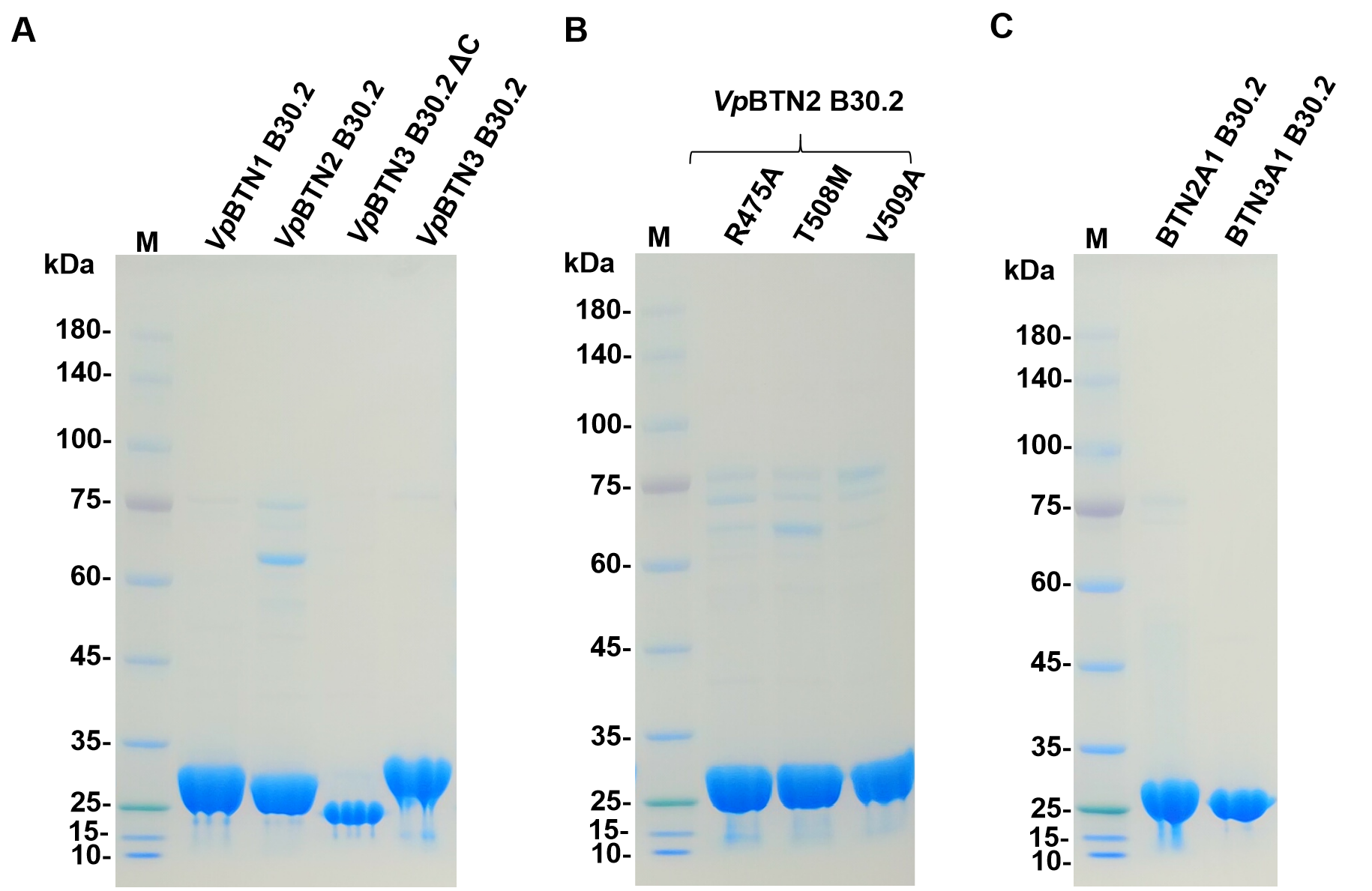
**

**Fig. S7 The SDS-PAGE results of BTN proteins in alpaca and human.**

1. The SDS-PAGE results of the B30.2 domains of *Vp*BTN1, *Vp*BTN2, *Vp*BTN3 ΔC and *Vp*BTN3. (B) The SDS-PAGE results for the three mutant proteins of the *Vp*BTN2 B30.2 domain. (C) The SDS-PAGE results of the B30.2 domains of BTN2A1 and BTN3A1.

**
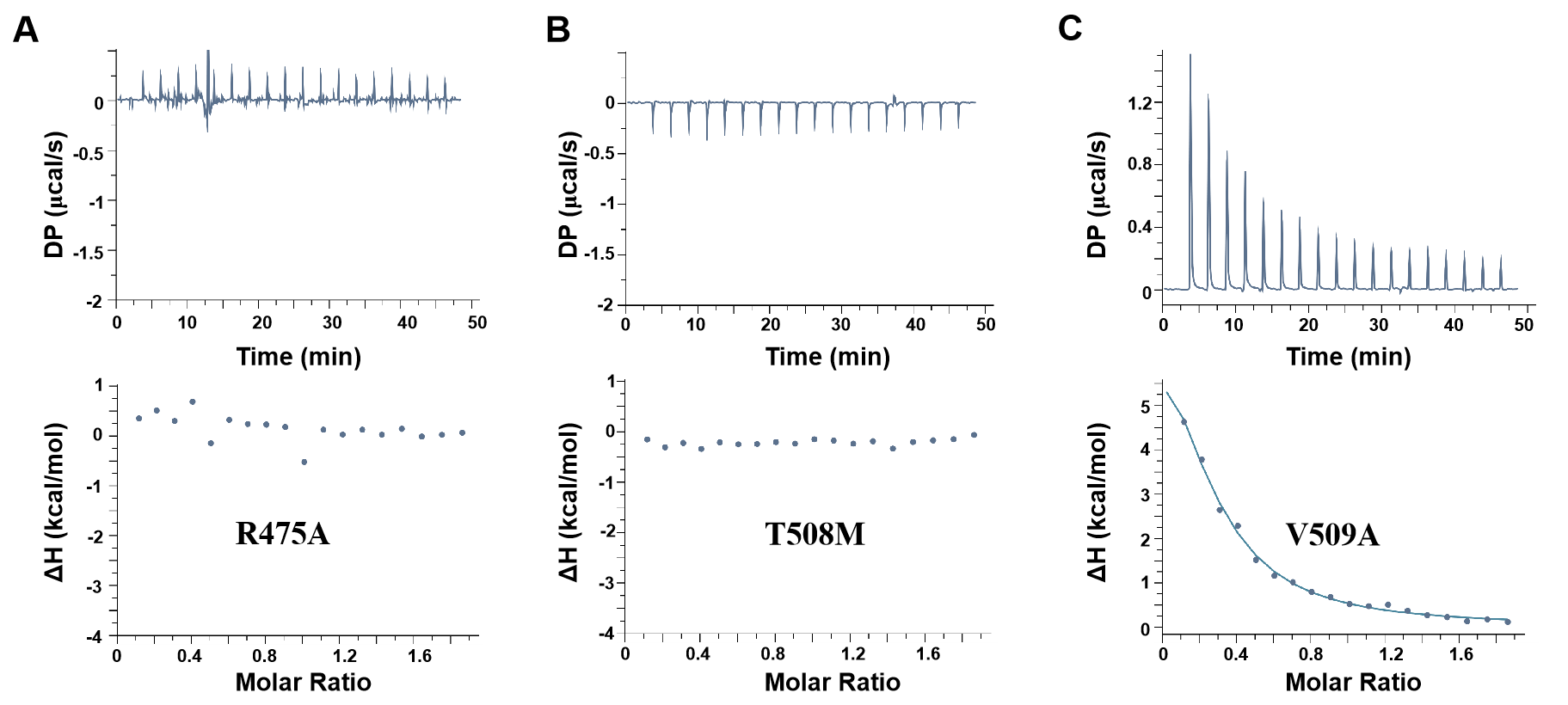
**

**Fig. S8 ITC results for three variants of *Vp*BTN2 B30.2**

(A) ITC result for *Vp*BTN2^R475A^ B30.2 domain binding to the *Vp*BTN3 B30.2 ΔC/DMASPP. (B) ITC result for *Vp*BTN2^T508M^ B30.2 domain binding to the *Vp*BTN3 B30.2 ΔC/DMASPP. (C) ITC result for *Vp*BTN2^V509A^ B30.2 domain binding to the *Vp*BTN3 B30.2 ΔC/DMASPP.

**
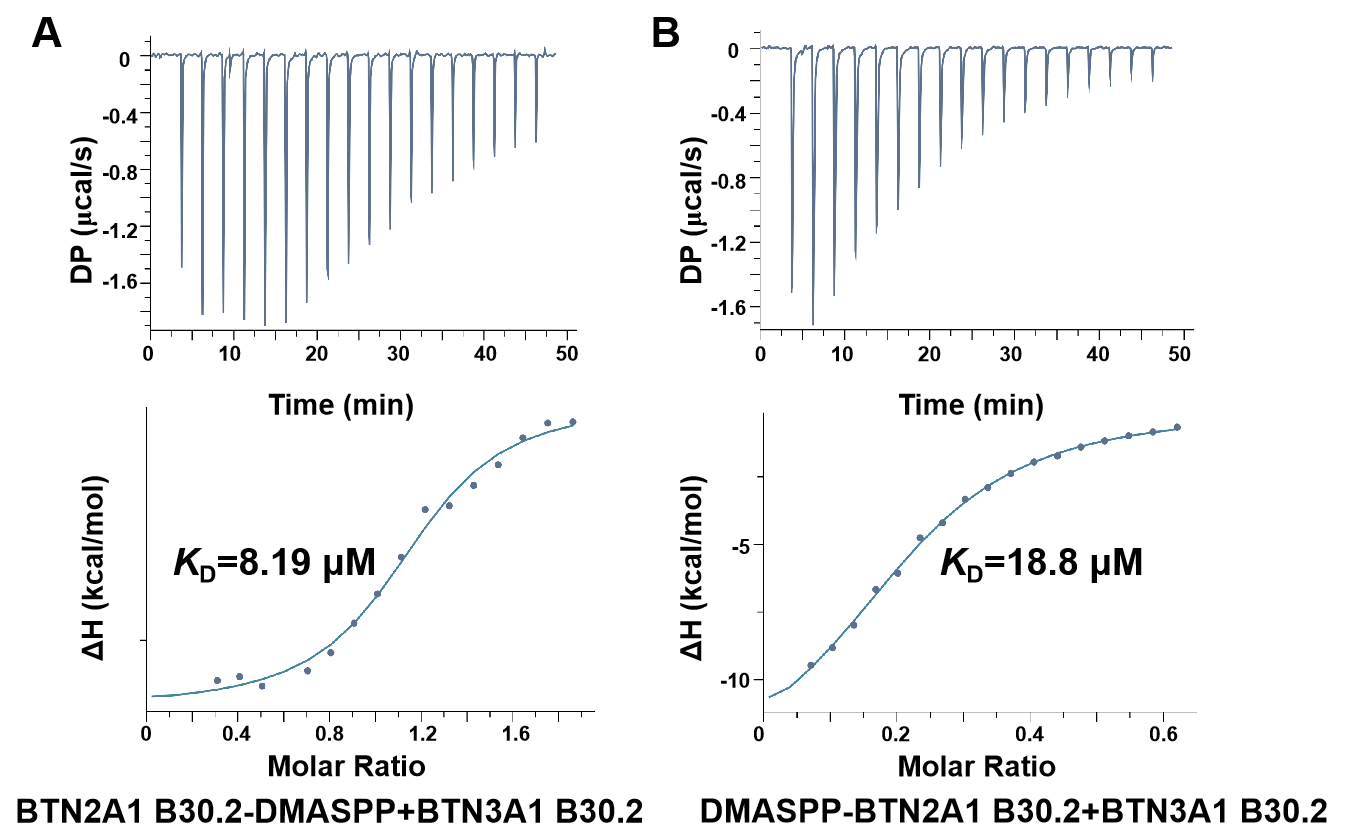
**

**Fig. S9 ITC results for human BTN3A1 and BTN2A1 B30.2 domains in the presence of DMASPP.**

ITC results shown that DMASPP promotes interaction between BTN2A1 and BTN3A1 B30.2 in the first titration mode (A) and in the second titration mode (B).

Table S1. X-ray data collection and refinement statistics.

|  | *Vp*BTN3 B30.2 ΔC/DMASPP | *Vp*BTN3 B30.2 ΔC/HMBPP-08 |
| --- | --- | --- |
| PDB code | 9LN2 | 9LNZ |
| **Data Collection** |  |  |
| Space group | *P*2_1_2_1_2_1_ | *P*2_1_2_1_2_1_ |
| Unit-cell |  |  |
| *a, b, c* (Å) | 43.86, 67.99, 134.31 | 43.79, 67.83, 134.81 |
| *α /β /γ* (°) | 90/90/90 | 90/90/90 |
| Resolution (Å) | 36.72-1.77(1.80-1.77) | 32.91-1.93 (1.96-1.93) |
| Unique reflections | 39950 (1878) | 30874 (1316) |
| Redundancy | 11.5 (4.7) | 5.0 (3.7) |
| Completeness (%) | 99.6 (96.5) | 99.3 (95.6) |
| Average I/σ (I) | 31.9 (5.8) | 19.3 (5.9) |
| Wilson B-factor | 16.5 | 17.8 |
| *R*_merge_^a^ (%) | 4.6 (16.8) | 5.0 (16.7) |
| *R*_pim_^b^ (%) | 1.3 (8.1) | 2.4 (9.4) |
| CC_1/2_ | 1.00 (0.98) | 1.00 (0.97) |
| **Refinement** |  |  |
| No. of reflections | 37867 (2646) | 29241 (2061) |
| *R*_work_^c^ (95% of data) | 15.7 (20.6) | 15.5 (17.7) |
| *R*_free_^d^ (5% of data) | 20.2 (26.6) | 20.0 (23.4) |
| RMSD bonds (Å) | 0.01 | 0.01 |
| RMSD angles (º) | 1.81 | 1.66 |
| Dihedral angles |  |  |
| Most favored (%) | 98.2 | 97.6 |
| Allowed (%) | 1.8 | 2.4 |
| Disallowed (%) | 0.0 | 0.0 |
| No. of non-H atoms/average B (Å^2^) |  |  |
| Protein | 3107/18.0 | 3118/18.5 |
| Water | 440/28.0 | 359/27.1 |
| ION (SO_4_^2-^) | 20/67.5 | 25/67.0 |
| Ligand | 14/27.9 DMASPP | 22/18.3 HMBPP-08 |

Values in parentheses are for the highest resolution shell.

^a^*R*_merge_ = Σ*_hkl_*Σi|Ii*(hkl*) − ⟨$\bar{\text{I}}$(*hkl*)⟩|/Σ*_hkl_*ΣiIi(*hkl*), in which the sum is over all the *i* measured reflections with equivalent miller indices *hkl*; ⟨I(*hkl*)⟩ is the averaged intensity of these *i* reflections, and the grand sum is over all measured reflections in the data set.

^b^*R*_pim_=Σ*_hkl_*$\sqrt{1/n-1}$Σi|Ii (*hkl*)-$\bar{\text{I}}$(*hkl*)|/Σ*_hkl_*Σi Ii(*hkl*)

^c^ *R*_work_ is the measure of the quality of the atomic model obtained from the crystallographic data.

^d^ *R*_free_ is equivalent to *R* value but is calculated for 5 % of the reflections chosen at random and omitted from the refinement process.

**Table S2. Mutagenesis oligonucleotides**

| Mutant | Sequence (5’ - 3’) |
| --- | --- |
| R475A_Forward | TGTCCCGCGTCACCCTTCTCTGGGCCCCTGAGGCCCTT |
| R475A_Reverse | GAAGGGTGACGCGGGACATGTGTAGATGTGAGATCTGT |
| T508M_Forward | GGGGTCATGGTGCCCGAGGGCGGCTTGGTCCTTCACAG |
| T508M_Reverse | CTCGGGCACCATGACCCCCTGGGCCCCTATAAATGCTG |
| V509A_Forward | GTCACGGCGCCCGAGGGCGGCTTGGTCCTTCACAGGGC |
| V509A_Reverse | GCCCTCGGGCGCCGTGACCCCCTGGGCCCCTATAAATG |
